# Interconversion of multiferroic domains and domain walls

**DOI:** 10.1038/s41467-021-22808-7

**Published:** 2021-05-12

**Authors:** E. Hassanpour, M. C. Weber, Y. Zemp, L. Kuerten, A. Bortis, Y. Tokunaga, Y. Taguchi, Y. Tokura, A. Cano, Th. Lottermoser, M. Fiebig

**Affiliations:** 1 Department of Materials, ETH Zurich, Zurich, Switzerland; 2 Department of Physics, ETH Zurich, Zurich, Switzerland; 3 Department of Advanced Materials Science, University of Tokyo, Kashiwa, Japan; 4 RIKEN Center for Emergent Matter Science (CEMS), Wako, Japan; 5 Department of Applied Physics, University of Tokyo, Tokyo, Japan; 6 Univ. Grenoble Alpes, CNRS, Grenoble INP, Institut Néel, Grenoble, France

**Keywords:** Ferroelectrics and multiferroics, Magnetic properties and materials, Phase transitions and critical phenomena

## Abstract

Systems with long-range order like ferromagnetism or ferroelectricity exhibit uniform, yet differently oriented three-dimensional regions called domains that are separated by two-dimensional topological defects termed domain walls. A change of the ordered state across a domain wall can lead to local non-bulk physical properties such as enhanced conductance or the promotion of unusual phases. Although highly desirable, controlled transfer of these properties between the bulk and the spatially confined walls is usually not possible. Here, we demonstrate this crossover by confining multiferroic Dy_0.7_Tb_0.3_FeO_3_ domains into multiferroic domain walls at an identified location within a non-multiferroic environment. This process is fully reversible; an applied magnetic or electric field controls the transformation. Aside from expanding the concept of multiferroic order, such interconversion can be key to addressing antiferromagnetic domain structures and topological singularities.

## Abstract (web summary)

Domains and domain walls can have distinctively different physical properties. Here, the authors show how to transfer domains into domain walls and vice versa while maintaining their physical properties. Thereby the authors tune a multiferroic state between three and two dimensions.

## Introduction

Recently, the interests in ferroic materials with magnetic or electric order evolved from the spatially expanded bulk domains towards the spatially confined domain walls. As inherent inhomogeneity, domain walls are a source of specific phenomena that are forbidden in the uniform interior of the corresponding domains^1,2^. Examples are the occurrence of magnetization, polarization, magnetoelectric coupling, (super-)conductivity, memory effects, or a change of melting temperature^3–15^. Hence, domain walls may be regarded as additional state of a material, virtually a world in its own self and often discussed without reference to the surrounding bulk phase.

It would be very desirable if the dimensional limitation of these phenomena could be overcome. For example, one could consider the confinement of a multifunctional bulk state into domain walls where they could establish a form of rewritable electromagnetic circuits^5,16^. Reversely, the domain walls may seed the recovery of the original bulk state, acting as its memory^10,17^.

A class of materials in which the transition from bulk domains to domain walls could be virtually continuous are compounds with phase transitions, in which the symmetry of the domains and domain walls coincide crosswise on either sides of the phase boundary. In such materials, domain walls can in principle gradually transform into domains, and vice versa, across a first-order phase transition. This concept was discussed in theoretical physics^18–20^, and systems showing a behavior consistent with this concept were reported^21,22^. Conclusive observation of a smooth, deterministic transfer of a domain wall into a domain, however, and, in particular, the reversible interconversion between domains and domain walls have not been presented. Notably, the practical consequences of such interconversion for the physical properties and functionalities of materials were never debated.

We demonstrate this interconvertibility and apply it to tune a multiferroic state between three and two dimensions in a controlled way. Specifically, we confine a multiferroic bulk state into a multiferroic domain wall at an identified location within a non-multiferroic environment. We act on this spatially confined magnetoelectric object with magnetic or electric fields and evidence the presence of a switchable magnetization and polarization in the wall. We furthermore employ the fields to transfer the multiferroic domain wall back into a multiferroic bulk state. We then discuss the general occurrence and benefits of an ordered state with controllable transfer in between spatial expansion and spatial confinement.

## Results and discussion

For the reasons given below, we choose the rare-earth orthoferrite Dy_0.7_Tb_0.3_FeO_3_ (see elsewhere^23^ about sample preparation) as our model system. Multiferroicity occurs at 2.65 K, caused by simultaneous antiferromagnetic ordering of the rare-earth and iron spin systems, coupled by exchange striction^23^. An electric polarization *P*_s_ = 0.12 μC cm^−2^ coexists with a Dzyaloshinskii–Moriya-type weak magnetization *M*_s_ = 0.15 *μ*_B_ per formula unit, both oriented along the *c*-axis. Subsequently, at *T*_C_ ≃ 2.3 K a first-order spin reorientation of the iron sublattice from the multiferroic to an antiferromagnetic phase with *M*_s_, *P*_s_ = 0 occurs. The simplest possible form to describe such a transition in between two magnetic phases *M*1 and *M*2 is the phenomenological effective Hamiltonian^19,20^

1 $$H=A| \nabla \theta {| }^{2}+{K}_{1}{\sin }^{2}\theta +{K}_{2}{\sin }^{4}\theta .$$

**Fig. 1:**
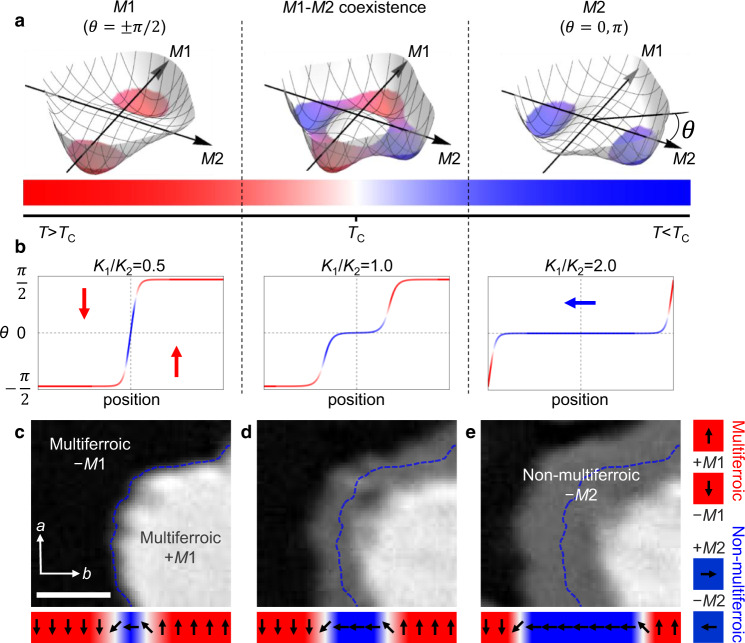
Interconversion of domains and domain walls. **a** Temperature dependence of the free-energy landscape for the first-order phase transition of Eq. (1) with *θ* as orientation of the order parameter. **b** Evolution of domains and domain walls across the *M*1 ↔ *M*2 phase transition. Domain walls expand into domains and domains are confined into domain walls, with an intermediate regime where the *M*1 and *M*2 phases coexist. *K*_1_ and *K*_2_ are the magnetocrystalline anisotropy parameters of Eq. (1). Blue and red arrows depict the orientation *θ* of the order parameter. **c**–**e** Upon temperature decrease, a domain wall (dashed blue line) separating opposite multiferroic domains in c expands and transforms to a domain of the non-multiferroic phase in **c** and **d**. Faraday rotation (FR) by *ϕ*_FR_ shows the *M*1 domains as dark (*ϕ*_FR_ < 0) and bright (*ϕ*_FR_ > 0) regions and the *M*2 domains (*ϕ*_FR_ = 0) as gray area. Scale bar, 100 μm.

As depicted in Fig. 1, the angle *θ* distinguishes the domain states on the two sides of the *M*1 ↔ *M*2 phase transition. *K*_1_ and *K*_2_ are magnetocrystalline anisotropy parameters. *K*_1_ = 0 and *K*_1_ = −2*K*_2_ set the limits of the phase-coexistence region of *M*1 and *M*2. The gradient term with *A* as exchange constant further determines the order-parameter rotation across the domain walls.

In Dy_0.7_Tb_0.3_FeO_3_, *M*1 and *M*2 are associated with the multiferroic (weakly ferromagnetic) and non-multiferroic (purely antiferromagnetic) phases, respectively, with *θ*_±*M*1_ = +*π*/2, −*π*/2 and *θ*_±*M*2_ = 0, *π*. Figure 1b depicts the evolution according to Eq. (1) of a configuration starting with two well-defined domains outside the coexistence region and separated by a single domain wall^18^. By entering the *M*1/*M*2 coexistence region, the domain wall widens and transforms into a domain of the other phase. Conversely, the initial domain shrinks and eventually transforms into a domain wall separating the domains on the opposite side of the coexistence region. Hence, the first-order phase transition between the multiferroic *M*1 and the non-multiferroic *M*2 phase in Dy_0.7_Tb_0.3_FeO_3_ should in principle allow us to reversibly convert a multiferroic domain into a multiferroic domain wall in a non-multiferroic environment.

We first verify the theoretically predicted transformation of a domain wall into a domain^18^ experimentally. In Dy_0.7_Tb_0.3_FeO_3_, we can exploit the magnetization of the multiferroic phase to distinguish the *M*1 and *M*2 domain states by spatially resolved real-time imaging experiments, using the magneto-optical Faraday effect as magnetization probe. Furthermore, Dy_0.7_Tb_0.3_FeO_3_ exhibits a relatively broad region of phase coexistence. This gives us ample time to image the phase coexistence while tuning the balance between the competing multiferroic and non-multiferroic phases.

Figure 1c–e shows sequential Faraday images and the associated order-parameter distribution of a *c*-oriented Dy_0.7_Tb_0.3_FeO_3_ sample cooled across the *M*1 ↔ *M*2 transition. We refrain from quantifying *K*_1_ and *K*_2_ since their absolute values are not relevant for our discussion. In Fig. 1c we see a ± *M*1 domain pair at *T* > *T*_C_. Because of the opposite direction of magnetization and, hence, Faraday rotation, the domains appear as bright and dark regions. Figure 1d, e show the same region about 4 and 8 min after cooling the sample to *T* ≲ *T*_C_. A gray stripe centers at the domain-wall position of Fig. 1c that widens with time. Its zero Faraday rotation identifies it as antiferromagnetic region. Below *T*_*C*_, this is the dominating phase and, starting from the original +*M*1/−*M*1 domain wall, we perceive the homogeneous expansion of this wall into an *M*2 domain engulfing the sample as time progresses. Note that this expansion of the non-multiferroic phase occurs in a deterministic way, starting uniformly from the center of the domain wall of the multiferroic phase. This deterministic behavior is in contrast to the needle-like growth of domains of the emergent phase at domain walls of the original phase^21^ or to the nucleation on random defects as in most first-order phase transitions^24^. Defects do not play a role in our case as we experimentally verify in Supplementary Note 3. Furthermore, the deterministic expansion requires an order-to-order transition, whereas order-to-disorder transitions into phase coexistence^25^ are not covered by Eq. (1) and must have a different origin^18^.

**Fig. 2:**
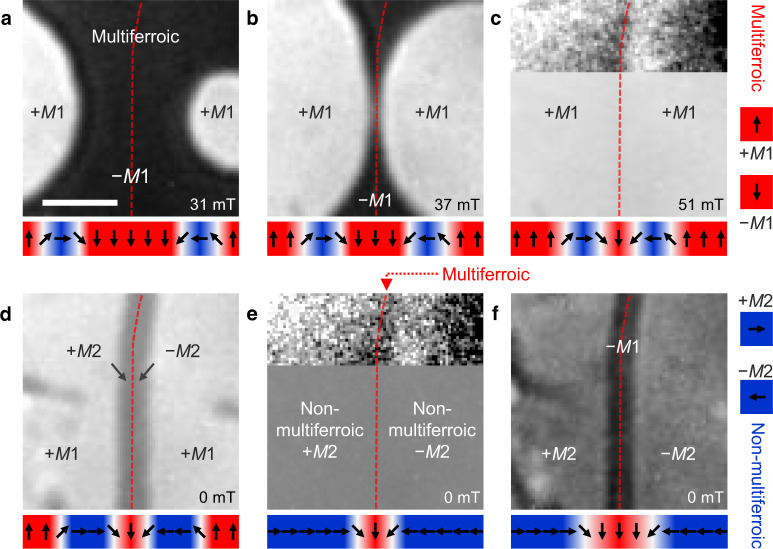
Transformation of a multiferroic bulk domain into a multiferroic domain wall in a non-multiferroic environment. Panels **a**–**f** show the same area, using the same gray scale (except in the insets of **c** and **e**, which are rescaled to minimum = black, maximum = white). Arrows indicate the order-parameter orientation (see Fig. 1a) for the horizontal succession of phases in the associated images. **a**–**c** Starting from a single-domain state (−*M*1, dark), two opposite multiferroic domains (+*M*1, bright) are generated by application of an out-of-plane magnetic field *H*. The +*M*1 domains grow until they meet along the red dashed line. Here, the two domain walls in **a** and **b** have merged into a single topological object, which becomes visible in the inset. **d**, **e** Setting *T* = 1.8 K at *H* = 0 returns the sample to the non-multiferroic *M*2 state. The wall separating the +*M*1 domains in **c** expands into opposite *M*2 domains (gray) separated by a multiferroic domain wall that becomes visible in the inset. **f** Heating the sample to 2.3 K lets the multiferroic domain wall in **e** expand into a multiferroic −*M*1 bulk domain. Scale bar, 100 μm.

Now we employ this deterministic aspect for confining a multiferroic domain into a multiferroic domain wall at an identified location within a non-multiferroic environment. We begin at *T* > *T*_C_ with a −*M*1 domain sandwiched between +*M*1 domains (Fig. 2a). In a magnetic field we enlarge the +*M*1 domains (Fig. 2b) until they meet (Fig. 2c). After cooling the sample to *T* ≲ *T*_C_, the antiferromagnetic state emerges from the spatially confined object at the meeting point of the +*M*1 domains (Fig. 2d) and grows with time until it has filled the entire field of view (Fig. 2e).

**Fig. 3:**
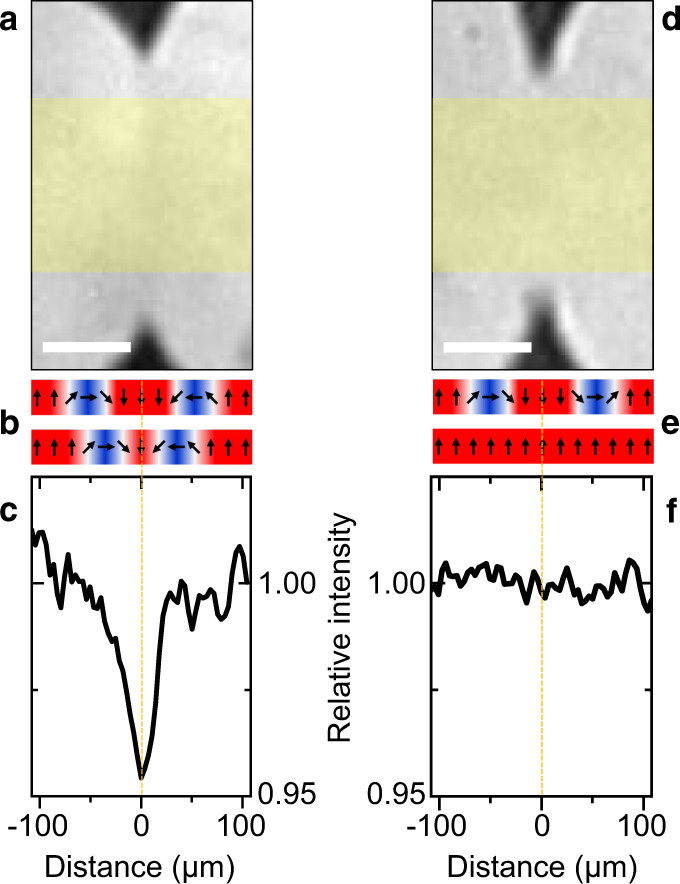
Non-coalescing and coalescing multiferroic domains. **a** Meeting of two multiferroic +*M*1 domains obtained following the procedure as in Fig. 2a–c. The sense of rotation of the order parameter across the +*M*1 → −*M*1 domain wall is the same for both +*M*1 domains. **b** Order-parameter orientation (arrows) across the boundary of the +*M*1 walls before (top) and after (bottom) the meeting of the +*M*1 domains. **c** Horizontal intensity scan of the image in **a**, vertically averaged across the area hatched yellow. The intensity dip indicates the formation of a 360^∘^ domain wall between the +*M*1 domains. **d**–**f** Same as **a**–**c**, but with opposite sense of rotation of the order parameter across the +*M*1 → −*M*1 domain wall for the two +*M*1 domains. Here the +*M*1 domains coalesce into a single domain. Scale bars, 100 μm.

As we see in Fig. 3, there are two types of meetings between +*M*1 domains on a −*M*1 background. As graphically illustrated in the Supplementary Note 4, they reflect the two types of domain walls a multiferroic domain is expected to exhibit, namely +*M*1 → −*M*1 walls with either clockwise or counterclockwise rotation of the order parameter across the wall^26^. When domain walls of the opposite type meet, the respective rotations cancel and the walls annihilate so that the meeting domains coalesce. Alternatively, for a meeting of the same type of domain walls, a 360^∘^ spin rotation occurs as sketched in Fig. 3b and Supplementary Note 4. This topologically protected object prevents the coalescence of the domains and is visible as brightness dip in Fig. 3c. Instead, it can seed a +*M*2 and a −*M*2 domain as sketched beneath Fig. 2d, e. Note that a magnetic field three times the value of the coercive field is required to destroy the 360^∘^ wall.

Hence, Fig. 2e shows a +*M*2 and a −*M*2 domain; according to Fig. 1a, they are separated by a multiferroic domain wall into which the multiferroic −*M*1 bulk state of Fig. 2a has been confined. This wall has been placed at the meeting point of the two former +*M*1 domains in Fig. 2c. For confirmation, we re-heat the sample to *T* ≳ *T*_C_; Fig. 2f shows that this reconverts the suspected multiferroic domain wall into a multiferroic −*M*1 domain. Note that even though the optical resolution is not sufficient to reveal the microscopic structure of the domain wall, we still can estimate its width to be about 1 μm through deconvolution of the optical signal as described in Supplementary Note 1. In complementary experiments on the closely related compound DyFeO_3_, we verify the value directly by magnetic force microscopy measurements in Supplementary Note 2. These measurements allow us to show the clear difference between the domain wall that we probe and the alternative case of a narrow domain.

**Fig. 4:**
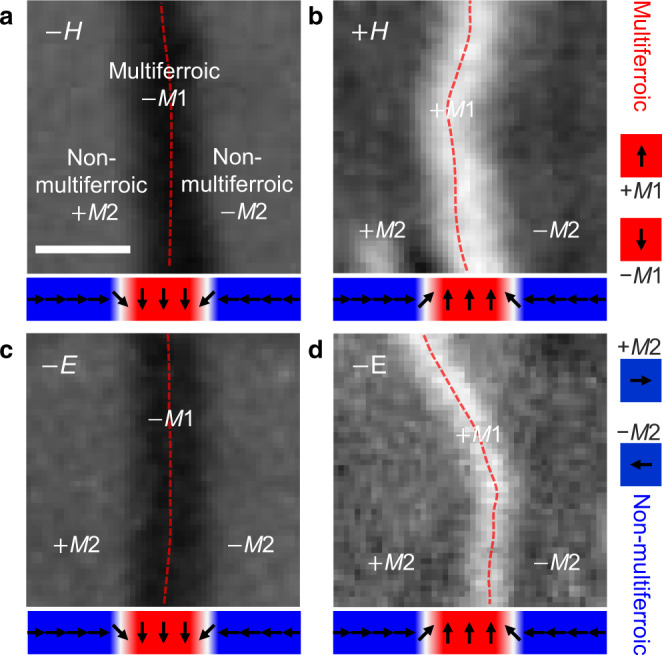
Magnetoelectric field control of multiferroic domain walls in a non-multiferroic environment. Using the same gray scale, **a**–**d** show equally sized regions on the same sample, yet imaged on different areas and in separate experiments. For experiments in **a**–**c**, a state as in Fig. 2e with +*M*2 and −*M*2 domains separated by a −*M*1-like domain wall was prepared while for **d**, opposite *M*2 domains are separated by a +*M*1-like domain wall. Magnetic and electric fields are applied as labeled, with the + sign indicating out-of-plane orientation. Beneath each image, the associated order-parameter orientation is sketched in analogy to Fig. 2. **a**, **b** A magnetic field ±*H* sets the magnetization of the multiferroic wall to ±*M*_s_ and expands it into a ±*M*1 multiferroic bulk domain with a 1:1 correspondence between the signs of *H* and *M*_s_. **c**, **d** An electric field ±*E* sets the polarization of the multiferroic wall to ±*P*_s_ and expands it into a multiferroic *M*1 bulk domain. Because of the involvement of three order parameters, the sign of *M*_s_ is not determined by the sign of *P*_s_(*E*) but rather by the history of the sample^27^. This is highlighted by **c** and **d**, where a field −*E* induces a +*M*1 or −*M*1 domain, respectively. Scale bar, 30 μm. *μ*_0_*H* = 0.4 T; *E* = 83.3 kV/cm.

To describe the wall as multiferroic, we furthermore have to show that we can act on it and affect its magnetization and electric polarization by an applied magnetic or electric field. This is also important for its technological utility. This is done in Fig. 4 which shows the response of a multiferroic domain wall in a non-multiferroic environment, generated as in Fig. 2e, to static *c*-oriented magnetic or electric fields. We see that both fields initiate the transfer of the wall back into a domain of the multiferroic phase, even though temperature-wise the material still favors the non-multiferroic phase. This is only possible because the wall has a magnetization *M*_s_ and a polarization *P*_s_ the respective applied field can act on to initiate the transfer. Furthermore, the transfer is energetically beneficial only if *M*_s_ or *P*_s_ points in the direction of the applied field. Since the field has triggered the transfer in all our experiments, the wall magnetization and polarization itself must be switchable, and hence set the direction of *M*_s_ or *P*_s_ of the expanding domain. Determination of the sign of *M*_s_ by the direction of the magnetic field is evident from Fig. 4a, b, whereas Fig. 4c shows that the electric field acts on the magnetization via magnetoelectric coupling. (Note that because of the involvement of three order parameters in this coupling^27^, the sign of *M*_s_ is not determined by the sign of *P*_s_(*E*) but rather by the history of the sample, as comparison of Fig. 4c, d shows.) We thus see that despite the spatial confinement and the non-multiferroic environment, a wall as in Fig. 2e retains the magnetoelectric manipulability of the multiferroic bulk phase.

To conclude, we have experimentally demonstrated the conversion of a multiferroic bulk phase into a multiferroic domain wall at an identified location inside a non-multiferroic environment. The initial multiferroic hallmark properties, namely a magnetization and a polarization that are coupled and switched by an applied field, are retained by the wall. Magnetic or electric fields act on the wall and convert it back into a bulk multiferroic domain with a predetermined direction of magnetization or polarization.

The concept of interconversion can be expanded beyond multiferroics. First, there are plenty of first-order magnetic phase transitions fulfilling the rather basic conditions of Eq. (1) with CaFe_2_O_4_ as a very recent example to catch scientific attention^28^. Note that even if the region of phase coexistence may be narrower than in our case, possibly even too narrow for convenient experimental probing during the transition, a system will still be subject to the same dynamics as found in Dy_0.7_Tb_0.3_FeO_3_. Even non-magnetic types of order can display the spatial interconversion. For example, a (static) expansion of domain walls into domains with phase coexistence based on a chemical gradient rather than the first-order nature of a phase transition was observed in the ferroelectric-to-nonferroelectric transition in InMnO_3_^29^. Even organic materials may show a transition between bulk phases with neutral and ionic building blocks emerging out of an expanding singularity by progressive charge transfer^30,31^.

Second, our work expands the possibilities for functionalizing domain walls. Unlike in previous examples^14,15^, the magnetoelectric properties are not confined to the domain walls but can be expanded into the bulk when needed. The magnetoelectric properties of the walls may be employed to visualize or even tailor domain patterns in materials whose order is normally difficult to access, such as 180° antiferromagnets. Finally, our work provides a rare opportunity of deterministic nucleation in a first-order phase transition since the nucleating phase is seeded by the order and symmetry in domain walls rather than occurring on random defects.

## Methods

Single-crystals of Dy_0.7_Tb_0.3_FeO_3_ were grown by the floating-zone method under O_2_ atmosphere. Using back-reflection X-ray Laue diffractometry, thin *c*-oriented plates of 2.5 × 2.5 mm^2^ were cut, lapped, and polished with a silica slurry down to ~60–80 μm with optically flat surfaces on both sides. Temperature and magnetic field treatment was done in a liquid-helium-cooled magnetic-field cryostat (Oxford Spectromag 4000-10T). For Faraday imaging, a light-emitting diode with a central wavelength of 660 nm (Thorlabs M660L4) was used along with a monochromatic digital camera (The Imaging Source DMK 22BUC03) for image acquisition. Image processing is limited to adjustments of brightness, contrast and levels and homogeneously applied to the entire image, unless noted. The images of each figure were adjusted equally. For electric field measurements, 3-nm platinum electrodes were deposited on both surfaces of the specimen.

### Reporting summary

Further information on research design is available in the Nature Research Reporting Summary linked to this article.

## Supplementary information

Supplementary Information

Lasing Reporting Summary

## Data Availability

The data that support the findings of this study are available from the corresponding author on request.
