# Supplementary material for: Interconversion of multiferroic domains and domain walls
Source: Nat Commun. 2021 May 12;12:2755. doi: 10.1038/s41467-021-22808-7 (PMC8115534; doi:10.1038/s41467-021-22808-7)
Supplement: Supplementary file 1 — Supplementary Information [file 41467_2021_22808_MOESM1_ESM.pdf]

Supplementary Information:
Interconversion of multiferroic domains and domain walls

E. Hassanpour,[1,2,†] M. C. Weber,[1,†]* Y. Zemp,[1] L. Kuerten,[1] A. Bortis,[1] Y. Tokunaga,[3] Y. Taguchi,[4] Y. Tokura,[4,5] A. Cano,[1,6] Th. Lottermoser,[1] and M. Fiebig[1]

[1]*Department of Materials, ETH Zurich, Vladimir-Prelog-Weg 4, 8093 Zurich, Switzerland*
[2]*Department of Physics, ETH Zurich, John-von-Neumann-Weg 9, 8093 Zurich, Switzerland*
[3]*Department of Advanced Materials Science, University of Tokyo, Kashiwa 277-8561, Japan*
[4]*RIKEN Center for Emergent Matter Science (CEMS), Wako 351-0198, Japan*
[5]*Department of Applied Physics, University of Tokyo, Tokyo 113-8656, Japan*
[6]*Univ. Grenoble Alpes, CNRS, Grenoble INP, Institut Néel, 38042 Grenoble, France*

†equal contribution.

*mads.weber@mat.ethz.ch

1. Calculation of the domain-wall-width by deconvolution

In Fig. 2e a width of the multiferroic domain wall of about 1 μm can be derived from optical measurements with a resolution limit of about 15 μm. In this section we explain how the associated deconvolution works.

The magnetization of the wall is determined by Faraday rotation. The optical setup is adjusted such that the +*M1* and the −*M1* domains appear as bright and dark regions, respectively. The brightness of the antiferromagnetic *M2* phase with zero Faraday rotation is intermediate. For the investigation of a −*M1*-type wall, the −*M1*-intensity level is normalized to 0 and the *M2*-intensity level to 1. For an infinitely small optical resolution, the spatial intensity profile of a ferromagnetic wall in a non-ferromagnetic environment would then look like the orange graph in Fig. S1. The limited resolution of the optical measurement broadens this profile into the replica shown as the blue graph in Fig. S1. The integrated area under the orange and blue graphs (taking 1 as the base line) must be the same, however, because the total optical signal does not depend on the resolution. This allows us to deconvolute the measured data and calculate the true width of the domain wall. In doing so, we use FWHM values and assume Gaussian profiles for both the measured and the actual magnetic-wall profile. This may introduce a systematic error on the order of a few percent that we accept. Note the special case of a magnetic 360° domain wall analyzed in Figs. 2c and 3a. Here the deconvolution procedure is the same as for the ferromagnetic wall.

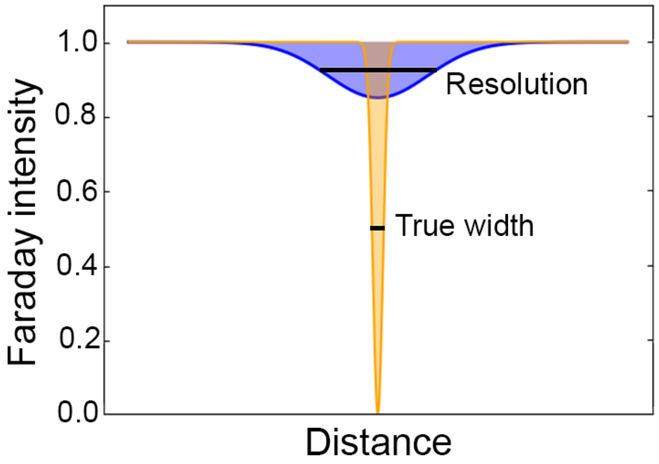

Figure S1: Calculating the domain-wall width. *Deconvolution*: Spatial intensity profile resulting from the magnetization of the domain wall. Orange and blue lines sketch the true wall profile and the measured optical-resolution-broadened profile, respectively. The shaded areas under the orange and blue curves must be the same (see text), which allows to derive the true domain-wall width.

2. Complementary MFM measurements on $DyFeO_3$

To complement the optical analyses in the main text by non-resolution-limited experiments, we performed magnetic force microscopy (MFM) measurements in an attocube attoLiquid low-temperature scanning probe microscope (SPM) setup with a spatial resolution of approximately 50 nm. Magnetic sensitivity was provided by PPP-MFMR hard magnetic AFM tips. At temperatures below 5 K, the stability of the SPM system is insufficient for resolving the multiferroic order of $Dy_{0.7}Tb_{0.3}FeO_3$. However, in $DyFeO_3$ the Fe spins are subject to the same type of transition between ferromagnetic and antiferromagnetic order as in $Dy_{0.7}Tb_{0.3}FeO_3$, yet at much higher temperature (~50 K)[1]. The material is not ferroelectric/multiferroic, but the domain walls in the antiferromagnetic state below ~50 K carry, as in $Dy_{0.7}Tb_{0.3}FeO_3$, a magnetic moment[2]. In magnetic terms, $DyFeO_3$ is a reasonable approximation to $Dy_{0.7}Tb_{0.3}FeO_3$, which gives us a good impression of the non-resolution-limited structure of the domain walls.

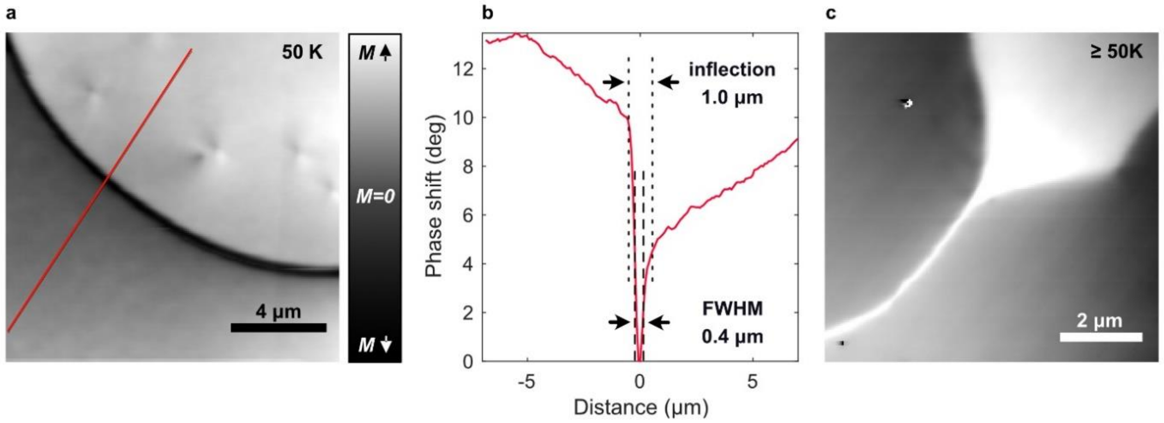

Figure S2: Ferromagnetic domain walls in $DyFeO_3$ by scanning probe microscopy. **a**, Spatially resolved MFM image of a down-magnetized ferromagnetic domain wall in an antiferromagnetic environment. The asymmetry between the two sides of the wall is an experimental artifact of the low-temperature MFM measurement. **b**, Cross-section of the domain wall along the red line in (a). The aforementioned asymmetry and the unknown magnetization profile of the wall lead to a systematic uncertainty in the determination of the domain-wall width. We find a value on the order of 0.4 to 1 μm. **c**, MFM image at the boundary between the ferromagnetic and the antiferromagnetic phase. An up-magnetized ferromagnetic domain shrinks to a ferromagnetic domain wall in an antiferromagnetic environment.

Figure S2 shows that ferromagnetic domain walls in a non-ferromagnetic environment are indeed observed in $DyFeO_3$. They can be up- or down-magnetized and, depending on the criterion applied to define the wall thickness, they exhibit a width between about 0.4 and 1 μm. In Fig. S2c we furthermore see that at the boundary between ferromagnetic and antiferromagnetic phase the domain wall expands from a line of constant width to an extending domain. The cross-section perpendicular to this line in Fig. S2b shows that the magnetization changes continuously. This continuous cross-sectional change distinguishes a ferromagnetic domain wall from a ferromagnetic domain. The latter would have to exhibit a region of uniform magnetization in its cross-section. Hence, the high-resolution MFM images confirm that we observe domain walls carrying a ferromagnetic moment rather than very thin ferromagnetic domains.

3. Domain nucleation

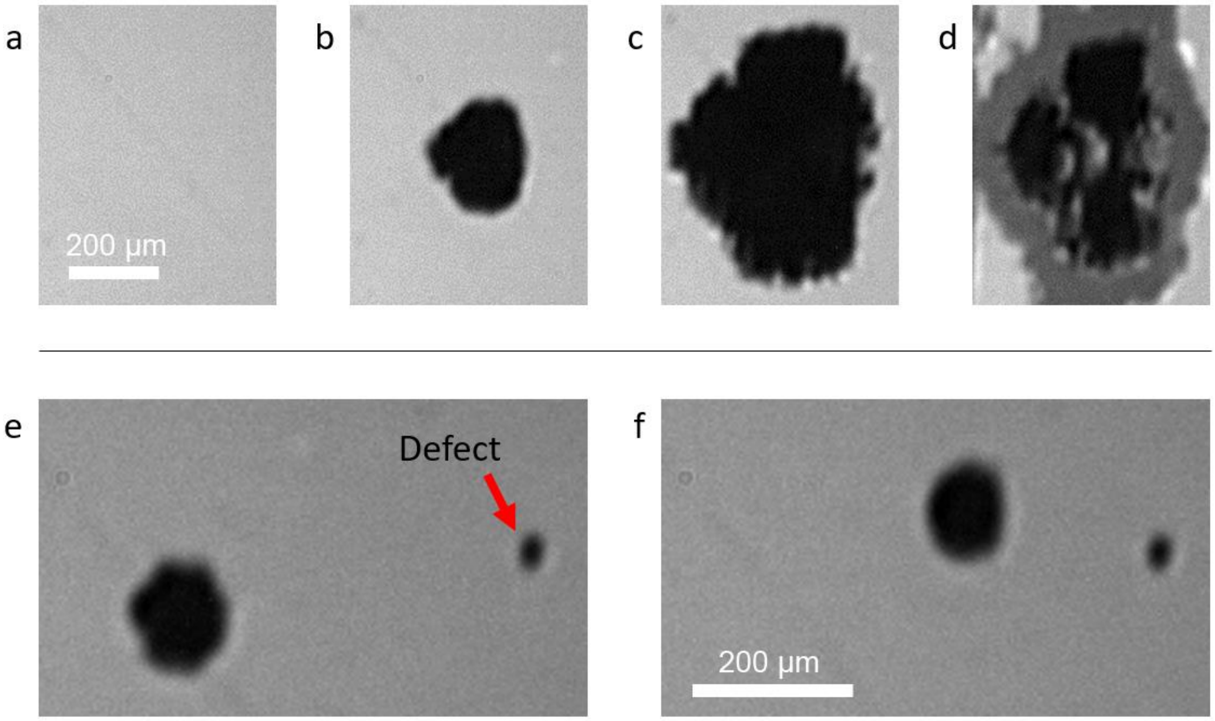

Figure S3: Nucleation of ferromagnetic domains in a magnetic field. a-c, Faraday-rotation microscopy images of the nucleation and growth of a down-magnetized domain within an up-magnetized environment in a magnetic field of increasing strength. **d**, Temperature decrease at zero magnetic field initiates the transfer of the domain wall separating the $+M1$ and $-M1$ regions into an antiferromagnetic domain. The size of the magnetic bubble in (c) is only determined by the time at which its expansion is stopped by setting the magnetic field to zero. Hence, the location at which the antiferromagnetic domain emerges out of the ferromagnetic domain wall is purely arbitrary and not guided by sample defects or other pinning effects. **e**, **f**, Magnetic-field-induced nucleation of a down-magnetized domain within an up-magnetized environment in consecutive magnetic-field cycles. The down-magnetized domain appears at different positions, showing that even their nucleation is not subject to defects or other pinning effects.

4. Helicity of the domain walls

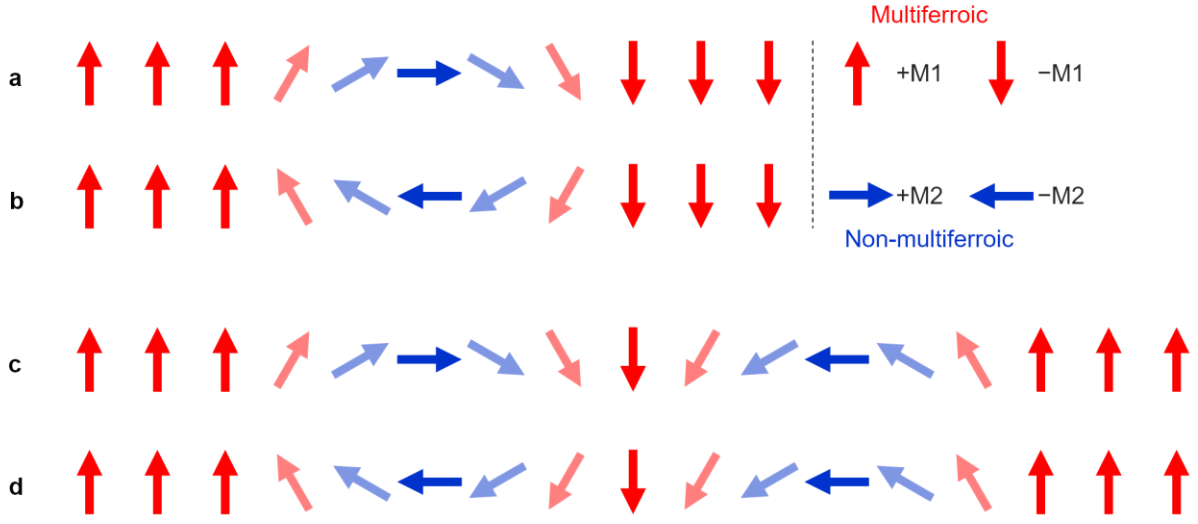

Figure S4: Helicity and meeting of domain walls. a,**b** The transition from a ferromagnetic +*M1* domain to a ferromagnetic –*M1* domain can occur as positive-helical or negative-helical rotation of the magnetization across the wall. The two types of domain walls are equivalent in energy and have therefore equal probability. **c**, Sketch of the meeting of two ferromagnetic domain walls with the same helicity in going from +*M1* to –*M1*. The 180° rotation of the magnetization across the walls add up to a 360° rotation which leads to the topological object discussed in Fig. 3a-c. **d**, Sketch of the meeting of two ferromagnetic domain walls with opposite helicity in going from +*M1* to –*M1*. The ±180° rotation of the magnetization across the walls add up to a zero. The domain walls annihilate each other as seen in Fig. 3d-e.

Supplementary references

1. Cao, S. *et al.* Tuning the Weak Ferromagnetic States in Dysprosium Orthoferrite. *Sci. Rep.* **6**, 37529 (2016).
2. Zalesski, A. V, Savvinov, A. M., Zheludev, I. S. & Ivashchenko, A. N. NMR of Fe^{57} nuclei and reorientation of spins in domains and domain walls of $ErFeO_3$ and $DyFeO_3$ crystals. *JETP* **41**, 723–728 (1975).
