# Supplementary material for: Interconversion of multiferroic domains and domain walls
Source: Nat Commun. 2021 May 12;12:2755. doi: 10.1038/s41467-021-22808-7 (PMC8115534; doi:10.1038/s41467-021-22808-7)
Supplement: Supplementary file 2 — Lasing Reporting Summary [file 41467_2021_22808_MOESM2_ESM.pdf]

nature research

Corresponding author(s): Mads Weber

Lasing Reporting Summary

Nature Research wishes to improve the reproducibility of the work that we publish. This form is intended for publication with all accepted papers reporting claims of lasing and provides structure for consistency and transparency in reporting. Some list items might not apply to an individual manuscript, but all fields must be completed for clarity.

For further information on Nature Research policies, including our data availability policy, see Authors & Referees.

ü Experimental design

Please check: are the following details reported in the manuscript?

1. Threshold

Plots of device output power versus pump power over a wide range of values indicating a clear threshold

☐ Yes
☒ No

It does not apply to our manuscript.

2. Linewidth narrowing

Plots of spectral power density for the emission at pump powers below, around, and above the lasing threshold, indicating a clear linewidth narrowing at threshold

☐ Yes
☒ No

It does not apply to our manuscript.

Resolution of the spectrometer used to make spectral measurements

☐ Yes
☒ No

It does not apply to our manuscript.

3. Coherent emission

Measurements of the coherence and/or polarization of the emission

☐ Yes
☒ No

It does not apply to our manuscript.

4. Beam spatial profile

Image and/or measurement of the spatial shape and profile of the emission, showing a well-defined beam above threshold

☐ Yes
☒ No

It does not apply to our manuscript.

5. Operating conditions

Description of the laser and pumping conditions
Continuous-wave, pulsed, temperature of operation

☐ Yes
☒ No

It does not apply to our manuscript.

Threshold values provided as density values (e.g. W cm^{-2} or J cm^{-2}) taking into account the area of the device

☐ Yes
☒ No

It does not apply to our manuscript.

6. Alternative explanations

Reasoning as to why alternative explanations have been ruled out as responsible for the emission characteristics
e.g. amplified spontaneous, directional scattering; modification of fluorescence spectrum by the cavity

☐ Yes
☒ No

It does not apply to our manuscript.

7. Theoretical analysis

Theoretical analysis that ensures that the experimental values measured are realistic and reasonable
e.g. laser threshold, linewidth, cavity gain-loss, efficiency

☐ Yes
☒ No

It does not apply to our manuscript.

8. Statistics

Number of devices fabricated and tested

☐ Yes
☒ No

It does not apply to our manuscript.

Statistical analysis of the device performance and lifetime (time to failure)

☐ Yes
☒ No

It does not apply to our manuscript.
